# 5,11,17,23-Tetra-*tert*-butyl-25,26,27,28-tetra­kis[2-(2-chloro­ethoxy)eth­oxy]-2,8,14,20-tetra­sulfonyl­calix[4]arene

**DOI:** 10.1107/S1600536809002591

**Published:** 2009-01-28

**Authors:** Ling Hu, Yang Liu, Jiang-Ping Ma, Dian-Shun Guo

**Affiliations:** a Department of Chemistry, Shandong Normal University, Jinan 250014, People’s Republic of China

## Abstract

Mol­ecules of the title compound, C_56_H_76_Cl_4_O_16_S_4_, have crystallographic *C*_2_ symmetry and adopt a 1,3-alternate conformation where the four –OCH_2_CH_2_OCH_2_CH_2_Cl groups are located alternately above and below the virtual plane (*R*) defined by the four bridging S atoms. The dihedral angles between the plane (*R*) and the phenolic rings are 72.85 (7) and 74.57 (7)°. An unusual 24-membered macrocyclic ring is formed in the crystal structure with an array of eight intra­molecular C—H⋯O hydrogen bonds between the ether arm H atoms and the sulfonyl O atoms. In the supra­molecular structure, the mol­ecular components are linked into infinite zigzag one-dimensional chains by a combination of four inter­molecular C—H⋯O hydrogen bonds, forming *R*_2_^2^(13), *R*_2_^2^(16), *R*_2_^2^(21) and *R*_2_^2^(26) ring motifs. These chains are augmented into a wave-like two-dimensional network by weak C⋯O inter­actions. One *tert*-butyl group shows rotational disorder, and one CH_2_CH_2_Cl group is disordered over two orientations; the site-occupation factors are 0.756 (6) and 0.244 (6) for the two *tert*-butyl groups, and 0.808 (3) and 0.192 (3) for the two CH_2_CH_2_Cl units.

## Related literature

For general background on the chemistry of thia­calix[4]arene derivatives, see: Shokova & Kovalev (2003 ▶); Lhoták (2004 ▶); Morohashi *et al.* (2006 ▶). For related crystal structures, see: Mislin *et al.* (1998 ▶, 1999 ▶); Akdas *et al.* (1999 ▶, 2000 ▶); Lhoták *et al.* (2002 ▶); Horiuchi *et al.* (2007 ▶); Xu *et al.* (2008 ▶). For the synthesis of sulfonyl­calix[4]arene derivatives, see: Iki *et al.* (1998 ▶); Guo *et al.* (2007 ▶). For hydrogen-bond motifs, see: Bernstein *et al.* (1995 ▶). For C⋯O short contacts, see: Manoj *et al.* (2007 ▶). For atomic radii, see: Bondi (1964 ▶).
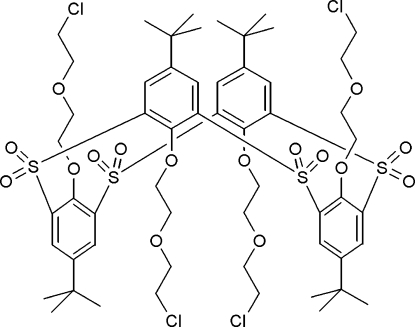


## Experimental

### 

#### Crystal data

C_56_H_76_Cl_4_O_16_S_4_
*M* *_r_* = 1275.21
Monoclinic, 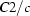

*a* = 22.496 (2) Å
*b* = 16.0372 (15) Å
*c* = 19.8646 (19) Å
β = 120.355 (1)°
*V* = 6184.1 (10) Å^3^
*Z* = 4
Mo *K*α radiation
μ = 0.39 mm^−1^
*T* = 173 (2) K
0.41 × 0.28 × 0.24 mm

#### Data collection

Bruker SMART CCD area-detector diffractometer
Absorption correction: multi-scan (*SADABS*; Bruker, 1999 ▶) *T*_min_ = 0.856, *T*_max_ = 0.912
15381 measured reflections
5442 independent reflections
4709 reflections with *I* > 2σ(*I*)
*R*_int_ = 0.022

#### Refinement

*R*[*F* ^2^ > 2σ(*F* ^2^)] = 0.058
*wR*(*F* ^2^) = 0.166
*S* = 1.09
5442 reflections
402 parameters
27 restraints
H-atom parameters constrained
Δρ_max_ = 1.30 e Å^−3^
Δρ_min_ = −0.80 e Å^−3^

### 

Data collection: *SMART* (Bruker, 1999 ▶); cell refinement: *SAINT* (Bruker, 1999 ▶); data reduction: *SAINT*; program(s) used to solve structure: *SHELXS97* (Sheldrick, 2008 ▶); program(s) used to refine structure: *SHELXL97* (Sheldrick, 2008 ▶); molecular graphics: *SHELXTL* (Sheldrick, 2008 ▶); software used to prepare material for publication: *SHELXTL*.

## Supplementary Material

Crystal structure: contains datablocks I, global. DOI: 10.1107/S1600536809002591/zl2171sup1.cif

Structure factors: contains datablocks I. DOI: 10.1107/S1600536809002591/zl2171Isup2.hkl

Additional supplementary materials:  crystallographic information; 3D view; checkCIF report

## Figures and Tables

**Table 1:** Hydrogen-bond geometry (Å, °)

*D*—H⋯*A*	*D*—H	H⋯*A*	*D*⋯*A*	*D*—H⋯*A*
C11—H11*A*⋯O1^i^	0.99	2.48	3.232 (4)	133
C11—H11*B*⋯O3	0.99	2.51	3.103 (4)	118
C20—H20*B*⋯O1^ii^	0.98	2.57	3.377 (5)	139
C21—H21*C*⋯O8^iii^	0.98	2.60	3.462 (6)	146
C25—H25*A*⋯O2	0.99	2.58	3.099 (4)	113
C25—H25*B*⋯O4	0.99	2.45	3.217 (4)	134

Symmetry codes: (i) 
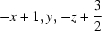
; (ii) 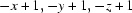; (iii) 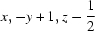.

**Fig. 1.**
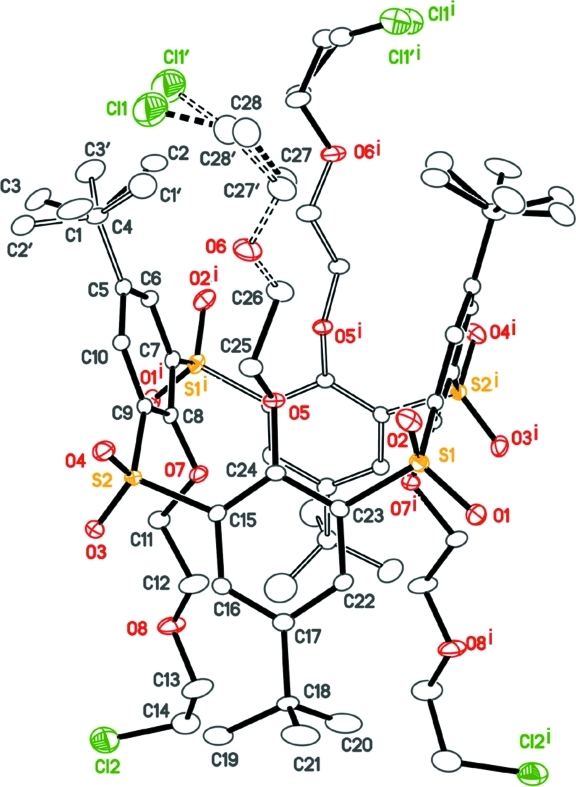
The molecular structure of the title compound with displacement ellipsoids drawn at the 30% probability level for non-H atoms. The disordered atoms are shown, but hydrogen atoms are omitted for clarity. [Symmetry code: (i) -x + 1, y, -z + 3/2].

**Fig. 2.**
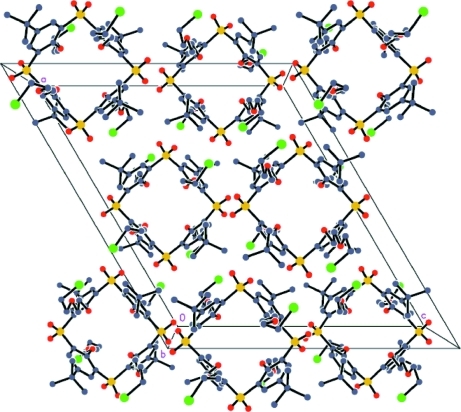
The crystal packing of the title compound viewed along the b axis. Hydrogen atoms and the minor disordered units are omitted for clarity.

**Fig. 3.**
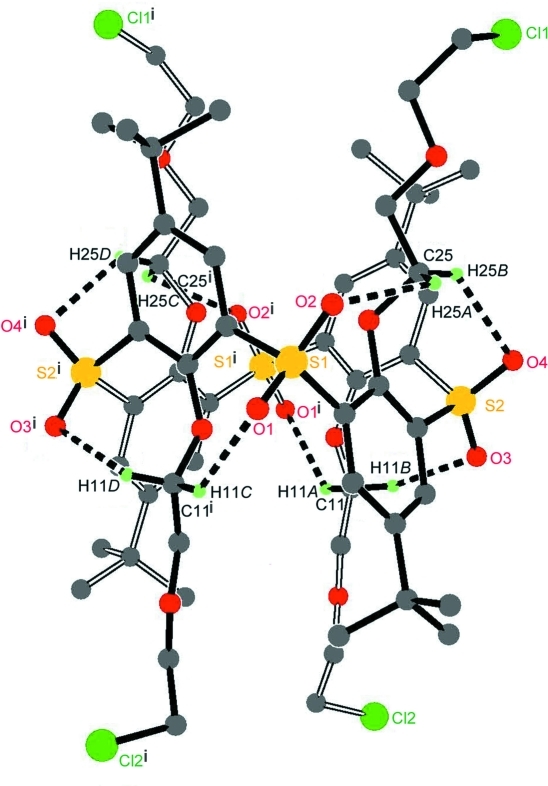
The array of intramolecular hydrogen-bonded rings of the title molecule viewed approximatively along the c axis. The minor disordered units and some hydrogen atoms are omitted for clarity. [Symmetry code: (i) -x + 1, y, -z + 3/2].

**Fig. 4.**
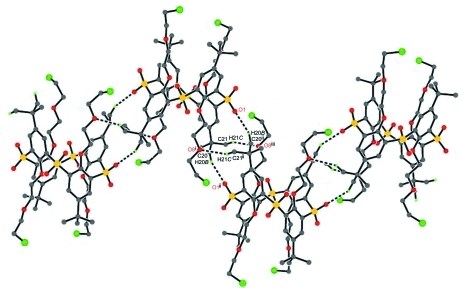
The hydrogen-bonded zigzag one-dimensional chains of the title molecule with R22(13), R22(16), R22(21) and R22(26) motifs. The minor disordered moieties and some hydrogen atoms are omitted for clarity. [Symmetry codes: (i) -x + 1, y, -z + 3/2; (ii) -x + 1, -y + 1, -z + 1; (iii) x, -y + 1, z - 1/2].

## supplementary crystallographic information

### Comment

Thiacalix[4]arenes have attracted considerable interest in recent years as useful scaffolds for highly organized ionophores (Shokova & Kovalev, 2003; Lhoták, 2004; Morohashi *et al.*, 2006; Guo *et al.*, 2007). Compared with classical calix[4]arenes, the presence of four bridging S atoms results in a differing complexation ability, and a diverse cavity and conformational behavior. Moreover, by virtue of the sulfide function, thiacalix[4]arenes can undergo unique transformations that are not applicable to the classical calix[4]arenes, the most important of which is oxidation to sulfinyl and sulfonyl functions. All four sulfide groups of thiacalix[4]arenes, for instance, can be easily converted to sulfones by a small excess amount of an oxidant such as hydrogen peroxide or sodium perborate in an organic acid solvent (Iki *et al.*, 1998; Mislin *et al.*, 1998). A number of crystal stuctures of sulfonyl derivatives of thiacalix[4]arenes (Mislin *et al.*, 1998; Akdas *et al.*, 2000; Lhoták *et al.*, 2002; Horiuchi *et al.*, 2007) have been described. We now present the crystal structure of a new sulfonyl derivative thiacalix[4]arene, 5,11,17,23-tetra-*tert*-butyl-25,26,27,28-tetrakis[2-(2-chloroethoxy)ethoxy]-2,8,14,20-tetrasulfonylcalix[4]arene.

The title sulfonylcalix[4]arene derivative is shown in Fig. 1. It was found to adopt a 1,3-alternate conformation with O atoms of the sulfones pointing outward. The main geometric parameters of the title molecule are comparable to those reported for the similar structures (Mislin *et al.*, 1998; Akdas *et al.*, 2000) and most bond lengths and angles are consistent with the values presented for 1,3-alternate-5,11,17,23-tetra-*tert*-butyl-25,26,27,28-tetrakismethyl-2,8,14,20-tetrasulfonylcalix[4]arene (Mislin *et al.*, 1998). The sulfonylcalix[4]arene shape of the title compound can be characterized by the values of the dihedral angles between the phenolic rings and the plane (*R*) defined by the four bridging S atoms. The dihedral angles between the plane (*R*) and the aromatic rings are 74.57 (7) and 72.85 (7)°, respectively. Actually, the title molecule has a pseudo 4-fold rotation-reflection (*S*_4_) axis. Consistent with this symmetry, the adjacent phenyl rings lie above and below the plane (*R*), and interplanar angles of the opposing aromatic rings are 34.31 (8) and 38.86 (14)°. The pseudo *S*_4_ symmetry also reasonably depicts the almost parallel orientation of the four ether arms above and below the plane (*R*). The separations between diametrically located ethereal O5 and O5^i^, O7 and O7^i^ [Symmetry code: (i) -*x* + 1, *y*, -*z* + 3/2] are 4.660 (4) and 4.347 (4) Å, respectively. In the crystal packing, 1,3-alternate molecules are packed along the *b* axis, forming a type of a beautiful nanotubular array extending in the *b* direction (Fig. 3). Such a packing was found in the cases of several 1,3-alternate thiaclix[4]arene derivatives (Akdas *et al.*, 1999, 2000; Guo *et al.*, 2007; Xu *et al.*, 2008).

Although no conventional hydrogen bonds are found, various intra- and intermolecular C—H···O hydrogen bonds exist in the crystal structure (Table 1). Interestingly, an unusual 24-membered macrocyclic ring is formed by an array of eight intramolecular C—H···O hydrogen bonds between the sulfonyl O atoms and the ether arm protons closer the phenolic rings, which stabilize the 1,3-alternate conformation (Fig. 4). In this macrocyclic ring, both O atoms of each sulfonyl group act as a hydrogen-bond acceptor, *via* H, to two C atoms belonging to both adjacent ether arms, respectively. A similar hydrogen bonding array was observed in the structure of the related compound *p*-*tert*-butyltetrasulfinylcalix[4]arene, however, it is formed with only four intramolecular O—H···O hydrogen bonds between the OH and SO groups (Mislin, *et al.*, 1999). On the other hand, in the supramolecular structure, infinite zigzag one-dimensional chains are generated by a combination of four intermolecular C—H···O hydrogen bonds, locally forming different ring motifs: two *R*_2_^2^(13), one *R*_2_^2^(16), two *R*_2_^2^(21), and one *R*_2_^2^(26) (Bernstein *et al.*, 1995), and making a distorted capsule at each link in the chain (Fig. 4). These motifs arise from atoms C20 and C21 at (*x*, *y*, *z*) and (-*x* + 1, -*y* + 1, -*z* + 1) in neighboring molecules that act as hydrogen-bond donors, respectively, *via* H20*B*, to atoms O1 at (-*x* + 1, -*y* + 1, -*z* + 1) and (*x*, *y*, *z*), *via* H21*C, *to atoms O8 at (*x*, -*y* + 1, *z* - 1/2) and (-*x* + 1, *y*, -*z* + 3/2). The zigzag chains are linked into wave-like two-dimensional networks by the C···O weak interactions (Manoj *et al.*, 2007) between C10^iv^ [symmetry code: (iv) – *x* + 3/2, – *y* + 3/2, –z + 2] and O3. The C10^iv^···O3 distance is 3.143 (4) Å, less than the sum of the van der Waals radii for C and O atoms (C = 1.70 Å, O = 1.52 Å; Bondi, 1964).

### Experimental

For the synthesis of the title compound, to a solution of 1,3-alternate-5,11,17,23-tetra-*tert*-butyl-25,26,27,28-tetrakis[2-(2-chloroethoxy)ethoxy]thiacalix[4]arene, prepared according to the published precedure (Guo *et al.*, 2007), (0.200 g, 0.174 mmol) in CHCl_3_ (10 ml) and CF_3_CO_2_H (1.50 ml) was added 30% H_2_O_2_ (0.90 ml, 7.840 mmol). The resulting mixture was stirred at 298 K for 50 h, and neutralized with a saturated aqueous solution of NaHCO_3_. The organic layer was separated and washed with brine, and dried over anhydrous MgSO_4_. Removal of the solvent under reduced pressure gave the title compound as a white solid (yield 95%) by recrystallization from CH_2_Cl_2_/CH_3_OH. ^1^H NMR (300 MHz, CDCl_3_): *δ* 8.38 (s, 8H), 4.58 (t, 8H, *J* = 5.67 Hz), 3.84 (t, 16H, *J* = 5.98 Hz), 3.71 (t, 8H, *J* = 5.83 Hz), 1.40 (s, 36H). IR (KBr pellets, cm^-1^): 1304, 1137. Single crystals of the title molecule suitable for X-ray diffraction analysis were obtained by slow evaporation of a solution in CH_2_Cl_2_ and CH_3_OH at 273 K.

### Refinement

All non-hydrogen atoms were refined with anisotropic displacement parameters. Hydrogen atoms attached to refined atoms were placed in geometrically idealized positions and refined using a riding model, with C—H = 0.93, 0.98 and 0.97 Å for aromatic, methylene and methyl H, respectively, and *U*_iso_(H) = 1.5*U*_eq_(C) for methyl H, and *U*_iso_(H) = 1.2*U*_eq_(C) for all other H atoms. In the title molecule, one of the symmetry-independent *tert*-butyl groups (C1—C4) shows rotational disorder, with refined site occupation factors of 0.756 (6):0.244 (6). The C—C bond lengths involving the disordered C atoms were restrained to be the same within a standard deviation of 0.02 Å, C—C distances refined to values between 1.472 and 1.5559 Å. The ADPs of C1', C2' and C3' were restrained to be isotropic within a standard deviation of 0.01 Å^2^. The atoms C27, C28 and Cl1 are disordered over two orientations, with refined site occupation factors of 0.808 (3):0.192 (3). The C—C, C—O and C—Cl bonds were restrained to be each the same within a standard deviation of 0.02 Å and refined to 1.453–1.461, 1.425–1.436 and 1.790–1.794 Å, respectively. The atoms C27', C28' and Cl1' were constrained to have the same ADPs as the atoms C27, C28 and Cl1.

### Crystal data

**Table:**

C_56_H_76_Cl_4_O_16_S_4_	*F*(000) = 2688
*M**_r_* = 1275.21	*D*_x_ = 1.370 Mg m^−^^3^
Monoclinic, *C*2/*c*	Mo *K*α radiation, λ = 0.71073 Å
*a* = 22.496 (2) Å	Cell parameters from 7613 reflections
*b* = 16.0372 (15) Å	θ = 2.2–28.1°
*c* = 19.8646 (19) Å	µ = 0.39 mm^−^^1^
β = 120.355 (1)°	*T* = 173 K
*V* = 6184.1 (10) Å^3^	Block, colourless
*Z* = 4	0.41 × 0.28 × 0.24 mm

### Data collection

**Table:**

Bruker SMART CCD area-detector diffractometer	5442 independent reflections
Radiation source: fine-focus sealed tube	4709 reflections with *I* > 2σ(*I*)
graphite	*R*_int_ = 0.022
φ and ω scans	θ_max_ = 25.0°, θ_min_ = 1.7°
Absorption correction: multi-scan (*SADABS*; Bruker, 1999)	*h* = −26→26
*T*_min_ = 0.856, *T*_max_ = 0.912	*k* = −16→19
15381 measured reflections	*l* = −23→23

### Refinement

**Table:**

Refinement on *F*^2^	Primary atom site location: structure-invariant direct methods
Least-squares matrix: full	Secondary atom site location: difference Fourier map
*R*[*F*^2^ > 2σ(*F*^2^)] = 0.058	Hydrogen site location: inferred from neighbouring sites
*wR*(*F*^2^) = 0.166	H-atom parameters constrained
*S* = 1.09	*w* = 1/[σ^2^(*F*_o_^2^) + (0.0863*P*)^2^ + 20.079*P*] where *P* = (*F*_o_^2^ + 2*F*_c_^2^)/3
5442 reflections	(Δ/σ)_max_ = 0.001
402 parameters	Δρ_max_ = 1.30 e Å^−^^3^
27 restraints	Δρ_min_ = −0.80 e Å^−^^3^

### Special details

**Table:**

Geometry. All e.s.d.'s (except the e.s.d. in the dihedral angle between two l.s. planes) are estimated using the full covariance matrix. The cell e.s.d.'s are taken into account individually in the estimation of e.s.d.'s in distances, angles and torsion angles; correlations between e.s.d.'s in cell parameters are only used when they are defined by crystal symmetry. An approximate (isotropic) treatment of cell e.s.d.'s is used for estimating e.s.d.'s involving l.s. planes.
Refinement. Refinement of *F*^2^ against ALL reflections. The weighted *R*-factor *wR* and goodness of fit *S* are based on *F*^2^, conventional *R*-factors *R* are based on *F*, with *F* set to zero for negative *F*^2^. The threshold expression of *F*^2^ > σ(*F*^2^) is used only for calculating *R*-factors(gt) *etc*. and is not relevant to the choice of reflections for refinement. *R*-factors based on *F*^2^ are statistically about twice as large as those based on *F*, and *R*- factors based on ALL data will be even larger.

### Fractional atomic coordinates and isotropic or equivalent isotropic displacement parameters (Å^2^)

**Table:**

	*x*	*y*	*z*	*U*_iso_*/*U*_eq_	Occ. (<1)
C5	0.63360 (14)	0.93392 (17)	0.93979 (16)	0.0206 (6)	
C6	0.58157 (15)	0.89626 (18)	0.94656 (16)	0.0216 (6)	
H6	0.5574	0.9283	0.9653	0.026*	
C7	0.56341 (14)	0.81334 (18)	0.92702 (15)	0.0206 (6)	
C8	0.59817 (14)	0.76368 (17)	0.90004 (15)	0.0198 (6)	
C9	0.65037 (14)	0.80187 (17)	0.89320 (15)	0.0200 (6)	
C10	0.66757 (14)	0.88537 (17)	0.91240 (16)	0.0202 (6)	
H10	0.7033	0.9093	0.9066	0.024*	
C11	0.60405 (18)	0.61541 (18)	0.92839 (18)	0.0298 (7)	
H11A	0.5787	0.6106	0.9570	0.036*	
H11B	0.6537	0.6229	0.9665	0.036*	
C12	0.5924 (2)	0.5401 (2)	0.8784 (2)	0.0429 (9)	
H12A	0.5460	0.5428	0.8313	0.051*	
H12B	0.6269	0.5386	0.8615	0.051*	
C13	0.5939 (3)	0.3938 (2)	0.8805 (3)	0.0513 (10)	
H13A	0.6368	0.3866	0.8787	0.062*	
H13B	0.5550	0.3982	0.8262	0.062*	
C14	0.5834 (2)	0.3196 (2)	0.9195 (3)	0.0529 (10)	
H14A	0.5730	0.2701	0.8855	0.063*	
H14B	0.5432	0.3299	0.9260	0.063*	
C15	0.64816 (14)	0.70631 (17)	0.77013 (16)	0.0191 (6)	
C16	0.65912 (14)	0.62388 (18)	0.75768 (17)	0.0220 (6)	
H16	0.6922	0.5919	0.8006	0.026*	
C17	0.62342 (15)	0.58698 (18)	0.68504 (17)	0.0242 (6)	
C22	0.57340 (15)	0.63497 (18)	0.62448 (17)	0.0245 (6)	
H22	0.5466	0.6110	0.5741	0.029*	
C23	0.56198 (15)	0.71742 (18)	0.63650 (17)	0.0217 (6)	
C24	0.60033 (14)	0.75573 (17)	0.70897 (16)	0.0196 (6)	
Cl2	0.65663 (6)	0.29815 (6)	1.01204 (7)	0.0613 (3)	
O1	0.46758 (12)	0.71591 (14)	0.49020 (12)	0.0331 (5)	
O2	0.53371 (11)	0.84535 (14)	0.54324 (13)	0.0309 (5)	
O3	0.72979 (11)	0.67345 (13)	0.91607 (12)	0.0303 (5)	
O4	0.75042 (11)	0.80237 (13)	0.86416 (13)	0.0295 (5)	
O7	0.57763 (11)	0.68433 (12)	0.87321 (11)	0.0252 (5)	
O8	0.59813 (14)	0.46765 (14)	0.92163 (14)	0.0395 (6)	
S1	0.50048 (4)	0.77435 (5)	0.55345 (4)	0.0239 (2)	
S2	0.70254 (4)	0.74481 (4)	0.86614 (4)	0.0216 (2)	
C1	0.7049 (3)	1.0578 (3)	0.9434 (4)	0.0496 (16)	0.756 (6)
H1A	0.7484	1.0276	0.9745	0.074*	0.756 (6)
H1B	0.7124	1.1174	0.9555	0.074*	0.756 (6)
H1C	0.6882	1.0492	0.8878	0.074*	0.756 (6)
C2	0.5861 (2)	1.0799 (3)	0.9178 (3)	0.0416 (13)	0.756 (6)
H2A	0.5975	1.1385	0.9326	0.062*	0.756 (6)
H2B	0.5518	1.0614	0.9311	0.062*	0.756 (6)
H2C	0.5672	1.0739	0.8615	0.062*	0.756 (6)
C3	0.6783 (3)	1.0349 (3)	1.0494 (3)	0.0467 (14)	0.756 (6)
H3A	0.7201	1.0013	1.0792	0.070*	0.756 (6)
H3B	0.6428	1.0156	1.0604	0.070*	0.756 (6)
H3C	0.6888	1.0936	1.0644	0.070*	0.756 (6)
C4	0.65239 (16)	1.02564 (18)	0.96255 (18)	0.0263 (7)	0.756 (6)
C1'	0.6356 (10)	1.0747 (10)	0.8892 (9)	0.056 (5)	0.244 (6)
H1E	0.5872	1.0658	0.8494	0.084*	0.244 (6)
H1F	0.6652	1.0557	0.8692	0.084*	0.244 (6)
H1D	0.6435	1.1343	0.9018	0.084*	0.244 (6)
C2'	0.7322 (6)	1.0296 (9)	1.0160 (9)	0.039 (4)	0.244 (6)
H2D	0.7461	1.0868	1.0346	0.059*	0.244 (6)
H2F	0.7540	1.0122	0.9862	0.059*	0.244 (6)
H2E	0.7467	0.9922	1.0606	0.059*	0.244 (6)
C3'	0.6215 (9)	1.0616 (10)	1.0060 (11)	0.052 (5)	0.244 (6)
H3E	0.5711	1.0590	0.9738	0.078*	0.244 (6)
H3D	0.6360	1.1198	1.0189	0.078*	0.244 (6)
H3F	0.6367	1.0298	1.0541	0.078*	0.244 (6)
C4'	0.65239 (16)	1.02564 (18)	0.96255 (18)	0.0263 (7)	0.244 (6)
C18	0.63795 (17)	0.49736 (19)	0.66984 (19)	0.0303 (7)	
C19	0.6904 (2)	0.4528 (2)	0.7460 (2)	0.0483 (10)	
H19A	0.6721	0.4501	0.7814	0.072*	
H19B	0.7339	0.4839	0.7709	0.072*	
H19C	0.6985	0.3962	0.7339	0.072*	
C20	0.5723 (3)	0.4473 (3)	0.6328 (3)	0.0730 (10)	
H20A	0.5541	0.4447	0.6683	0.110*	
H20B	0.5817	0.3907	0.6220	0.110*	
H20C	0.5383	0.4739	0.5838	0.110*	
C21	0.6712 (3)	0.5009 (3)	0.6204 (3)	0.0730 (10)	
H21A	0.6829	0.4443	0.6125	0.110*	
H21B	0.7132	0.5347	0.6468	0.110*	
H21C	0.6391	0.5259	0.5697	0.110*	
O5	0.58700 (10)	0.83690 (12)	0.71892 (11)	0.0222 (4)	
C25	0.63180 (16)	0.89918 (18)	0.71453 (19)	0.0285 (7)	
H25A	0.6423	0.8847	0.6732	0.034*	
H25B	0.6756	0.9033	0.7649	0.034*	
C26	0.59268 (19)	0.9801 (2)	0.6958 (2)	0.0406 (8)	
H26A	0.5534	0.9793	0.6416	0.049*	
H26B	0.5745	0.9888	0.7315	0.049*	
O6	0.63887 (13)	1.04475 (14)	0.70517 (16)	0.0440 (6)	0.808 (3)
C27	0.6023 (3)	1.1211 (3)	0.6756 (3)	0.0425 (13)	0.808 (3)
H27A	0.5774	1.1352	0.7033	0.051*	0.808 (3)
H27B	0.5681	1.1150	0.6194	0.051*	0.808 (3)
C28	0.6508 (5)	1.1877 (6)	0.6868 (4)	0.0571 (18)	0.808 (3)
H28A	0.6245	1.2387	0.6606	0.069*	0.808 (3)
H28B	0.6770	1.1713	0.6611	0.069*	0.808 (3)
Cl1	0.71051 (11)	1.21148 (10)	0.78691 (10)	0.0777 (6)	0.808 (3)
O6'	0.63887 (13)	1.04475 (14)	0.70517 (16)	0.0440 (6)	0.192 (3)
C27'	0.6214 (13)	1.1202 (16)	0.6602 (17)	0.0425 (13)	0.192 (3)
H27C	0.5721	1.1329	0.6394	0.051*	0.192 (3)
H27D	0.6285	1.1120	0.6154	0.051*	0.192 (3)
C28'	0.663 (3)	1.190 (3)	0.706 (2)	0.0571 (18)	0.192 (3)
H28C	0.6464	1.2395	0.6712	0.069*	0.192 (3)
H28D	0.7103	1.1793	0.7181	0.069*	0.192 (3)
Cl1'	0.6696 (5)	1.2238 (4)	0.7962 (5)	0.0777 (6)	0.192 (3)

### Atomic displacement parameters (Å^2^)

**Table:**

	*U*^11^	*U*^22^	*U*^33^	*U*^12^	*U*^13^	*U*^23^
C5	0.0221 (14)	0.0180 (14)	0.0176 (14)	−0.0005 (11)	0.0071 (12)	−0.0007 (11)
C6	0.0251 (15)	0.0190 (14)	0.0192 (14)	−0.0007 (11)	0.0100 (12)	−0.0019 (11)
C7	0.0229 (14)	0.0199 (14)	0.0151 (13)	−0.0034 (11)	0.0066 (12)	0.0010 (11)
C8	0.0243 (15)	0.0152 (14)	0.0126 (13)	−0.0018 (11)	0.0039 (11)	0.0009 (10)
C9	0.0217 (14)	0.0194 (14)	0.0138 (13)	0.0031 (11)	0.0051 (11)	−0.0002 (10)
C10	0.0213 (14)	0.0158 (14)	0.0194 (13)	−0.0008 (11)	0.0072 (11)	−0.0001 (11)
C11	0.0421 (19)	0.0162 (15)	0.0281 (16)	−0.0005 (13)	0.0156 (14)	0.0035 (12)
C12	0.077 (3)	0.0179 (17)	0.045 (2)	−0.0033 (16)	0.040 (2)	0.0011 (14)
C13	0.085 (3)	0.0215 (18)	0.056 (2)	−0.0058 (18)	0.042 (2)	−0.0071 (16)
C14	0.058 (3)	0.0266 (19)	0.071 (3)	−0.0038 (17)	0.031 (2)	−0.0027 (18)
C15	0.0207 (14)	0.0191 (14)	0.0193 (14)	−0.0015 (11)	0.0114 (12)	−0.0036 (11)
C16	0.0214 (14)	0.0183 (14)	0.0252 (15)	0.0022 (11)	0.0109 (12)	−0.0001 (11)
C17	0.0266 (15)	0.0209 (15)	0.0286 (15)	0.0021 (12)	0.0165 (13)	−0.0017 (12)
C22	0.0283 (16)	0.0217 (15)	0.0224 (15)	0.0020 (12)	0.0119 (13)	−0.0055 (11)
C23	0.0230 (14)	0.0212 (15)	0.0212 (14)	0.0051 (11)	0.0113 (12)	0.0014 (11)
C24	0.0229 (15)	0.0152 (14)	0.0239 (15)	0.0008 (11)	0.0143 (13)	−0.0003 (11)
Cl2	0.0629 (7)	0.0338 (5)	0.0756 (8)	−0.0030 (5)	0.0264 (6)	0.0063 (5)
O1	0.0391 (13)	0.0349 (13)	0.0200 (11)	0.0114 (10)	0.0110 (10)	−0.0027 (9)
O2	0.0374 (12)	0.0321 (12)	0.0307 (12)	0.0093 (10)	0.0227 (10)	0.0087 (9)
O3	0.0319 (12)	0.0228 (11)	0.0237 (11)	0.0081 (9)	0.0049 (9)	−0.0014 (8)
O4	0.0228 (11)	0.0255 (11)	0.0382 (12)	−0.0023 (9)	0.0141 (10)	−0.0090 (9)
O7	0.0332 (11)	0.0144 (10)	0.0211 (10)	−0.0039 (8)	0.0088 (9)	−0.0010 (8)
O8	0.0649 (17)	0.0150 (11)	0.0445 (14)	−0.0022 (10)	0.0321 (13)	−0.0002 (10)
S1	0.0294 (4)	0.0249 (4)	0.0178 (4)	0.0076 (3)	0.0122 (3)	0.0014 (3)
S2	0.0203 (4)	0.0175 (4)	0.0215 (4)	0.0023 (3)	0.0065 (3)	−0.0035 (3)
C1	0.057 (3)	0.025 (2)	0.089 (5)	−0.017 (2)	0.053 (3)	−0.020 (3)
C2	0.041 (3)	0.018 (2)	0.059 (3)	−0.0003 (19)	0.020 (2)	−0.003 (2)
C3	0.061 (4)	0.030 (3)	0.040 (3)	−0.013 (2)	0.018 (3)	−0.017 (2)
C4	0.0317 (16)	0.0156 (14)	0.0333 (17)	−0.0031 (12)	0.0176 (14)	−0.0046 (12)
C1'	0.065 (9)	0.034 (7)	0.052 (8)	−0.002 (6)	0.017 (6)	0.003 (6)
C2'	0.035 (7)	0.023 (6)	0.050 (7)	−0.007 (5)	0.013 (5)	−0.009 (5)
C3'	0.056 (8)	0.039 (7)	0.064 (8)	−0.007 (6)	0.033 (7)	−0.019 (6)
C4'	0.0317 (16)	0.0156 (14)	0.0333 (17)	−0.0031 (12)	0.0176 (14)	−0.0046 (12)
C18	0.0352 (17)	0.0196 (16)	0.0318 (17)	0.0063 (13)	0.0137 (14)	−0.0069 (12)
C19	0.071 (3)	0.0274 (19)	0.044 (2)	0.0165 (18)	0.027 (2)	0.0012 (16)
C20	0.086 (3)	0.0380 (18)	0.091 (3)	0.0217 (17)	0.042 (2)	−0.0082 (17)
C21	0.086 (3)	0.0380 (18)	0.091 (3)	0.0217 (17)	0.042 (2)	−0.0082 (17)
O5	0.0275 (11)	0.0138 (10)	0.0269 (11)	0.0020 (8)	0.0149 (9)	−0.0013 (8)
C25	0.0317 (17)	0.0172 (15)	0.0345 (17)	−0.0007 (12)	0.0153 (14)	0.0019 (12)
C26	0.039 (2)	0.0198 (16)	0.055 (2)	−0.0003 (14)	0.0174 (17)	0.0041 (15)
O6	0.0430 (14)	0.0185 (12)	0.0572 (16)	−0.0014 (10)	0.0157 (13)	0.0086 (11)
C27	0.042 (3)	0.0233 (19)	0.053 (3)	−0.002 (2)	0.018 (2)	0.012 (2)
C28	0.055 (5)	0.031 (2)	0.055 (5)	−0.011 (3)	0.006 (5)	0.012 (3)
Cl1	0.0839 (13)	0.0500 (8)	0.0731 (10)	−0.0158 (8)	0.0204 (9)	0.0028 (7)
O6'	0.0430 (14)	0.0185 (12)	0.0572 (16)	−0.0014 (10)	0.0157 (13)	0.0086 (11)
C27'	0.042 (3)	0.0233 (19)	0.053 (3)	−0.002 (2)	0.018 (2)	0.012 (2)
C28'	0.055 (5)	0.031 (2)	0.055 (5)	−0.011 (3)	0.006 (5)	0.012 (3)
Cl1'	0.0839 (13)	0.0500 (8)	0.0731 (10)	−0.0158 (8)	0.0204 (9)	0.0028 (7)

### Geometric parameters (Å, °)

**Table:**

C5—C10	1.381 (4)	C2—H2A	0.9800
C5—C6	1.383 (4)	C2—H2B	0.9800
C5—C4	1.534 (4)	C2—H2C	0.9800
C6—C7	1.388 (4)	C3—C4	1.523 (5)
C6—H6	0.9500	C3—H3A	0.9800
C7—C8	1.399 (4)	C3—H3B	0.9800
C7—S1^i^	1.781 (3)	C3—H3C	0.9800
C8—O7	1.367 (3)	C1'—H1E	0.9800
C8—C9	1.391 (4)	C1'—H1F	0.9800
C9—C10	1.393 (4)	C1'—H1D	0.9800
C9—S2	1.772 (3)	C2'—H2D	0.9800
C10—H10	0.9500	C2'—H2F	0.9800
C11—O7	1.455 (4)	C2'—H2E	0.9800
C11—C12	1.499 (5)	C3'—H3E	0.9800
C11—H11A	0.9900	C3'—H3D	0.9800
C11—H11B	0.9900	C3'—H3F	0.9800
C12—O8	1.411 (4)	C18—C20	1.506 (6)
C12—H12A	0.9900	C18—C21	1.507 (6)
C12—H12B	0.9900	C18—C19	1.545 (5)
C13—O8	1.414 (4)	C19—H19A	0.9800
C13—C14	1.503 (6)	C19—H19B	0.9800
C13—H13A	0.9900	C19—H19C	0.9800
C13—H13B	0.9900	C20—H20A	0.9800
C14—Cl2	1.775 (5)	C20—H20B	0.9800
C14—H14A	0.9900	C20—H20C	0.9800
C14—H14B	0.9900	C21—H21A	0.9800
C15—C16	1.390 (4)	C21—H21B	0.9800
C15—C24	1.393 (4)	C21—H21C	0.9800
C15—S2	1.777 (3)	O5—C25	1.453 (4)
C16—C17	1.381 (4)	C25—C26	1.505 (4)
C16—H16	0.9500	C25—H25A	0.9900
C17—C22	1.392 (4)	C25—H25B	0.9900
C17—C18	1.537 (4)	C26—O6	1.412 (4)
C22—C23	1.391 (4)	C26—H26A	0.9900
C22—H22	0.9500	C26—H26B	0.9900
C23—C24	1.392 (4)	O6—C27	1.425 (6)
C23—S1	1.778 (3)	C27—C28	1.461 (7)
C24—O5	1.373 (3)	C27—H27A	0.9900
O1—S1	1.437 (2)	C27—H27B	0.9900
O2—S1	1.432 (2)	C28—Cl1	1.790 (6)
O3—S2	1.433 (2)	C28—H28A	0.9900
O4—S2	1.434 (2)	C28—H28B	0.9900
S1—C7^i^	1.781 (3)	C27'—C28'	1.453 (18)
C1—C4	1.503 (5)	C27'—H27C	0.9900
C1—H1A	0.9800	C27'—H27D	0.9900
C1—H1B	0.9800	C28'—Cl1'	1.79 (2)
C1—H1C	0.9800	C28'—H28C	0.9900
C2—C4	1.560 (5)	C28'—H28D	0.9900
			
C10—C5—C6	117.1 (3)	C1—C4—C3	110.6 (4)
C10—C5—C4	122.1 (3)	C1—C4—C5	112.9 (3)
C6—C5—C4	120.7 (3)	C3—C4—C5	108.5 (3)
C5—C6—C7	122.4 (3)	C1—C4—C2	108.2 (4)
C5—C6—H6	118.8	C3—C4—C2	107.5 (3)
C7—C6—H6	118.8	C5—C4—C2	109.0 (3)
C6—C7—C8	120.7 (3)	H1E—C1'—H1F	109.5
C6—C7—S1^i^	115.6 (2)	H1E—C1'—H1D	109.5
C8—C7—S1^i^	123.5 (2)	H1F—C1'—H1D	109.5
O7—C8—C9	120.5 (3)	H2D—C2'—H2F	109.5
O7—C8—C7	122.4 (3)	H2D—C2'—H2E	109.5
C9—C8—C7	116.7 (3)	H2F—C2'—H2E	109.5
C8—C9—C10	121.9 (3)	H3E—C3'—H3D	109.5
C8—C9—S2	121.6 (2)	H3E—C3'—H3F	109.5
C10—C9—S2	116.4 (2)	H3D—C3'—H3F	109.5
C5—C10—C9	121.2 (3)	C20—C18—C21	112.8 (4)
C5—C10—H10	119.4	C20—C18—C17	110.0 (3)
C9—C10—H10	119.4	C21—C18—C17	108.5 (3)
O7—C11—C12	104.2 (2)	C20—C18—C19	107.9 (3)
O7—C11—H11A	110.9	C21—C18—C19	105.7 (3)
C12—C11—H11A	110.9	C17—C18—C19	111.9 (3)
O7—C11—H11B	110.9	C18—C19—H19A	109.5
C12—C11—H11B	110.9	C18—C19—H19B	109.5
H11A—C11—H11B	108.9	H19A—C19—H19B	109.5
O8—C12—C11	109.2 (3)	C18—C19—H19C	109.5
O8—C12—H12A	109.8	H19A—C19—H19C	109.5
C11—C12—H12A	109.8	H19B—C19—H19C	109.5
O8—C12—H12B	109.8	C18—C20—H20A	109.5
C11—C12—H12B	109.8	C18—C20—H20B	109.5
H12A—C12—H12B	108.3	H20A—C20—H20B	109.5
O8—C13—C14	110.3 (3)	C18—C20—H20C	109.5
O8—C13—H13A	109.6	H20A—C20—H20C	109.5
C14—C13—H13A	109.6	H20B—C20—H20C	109.5
O8—C13—H13B	109.6	C18—C21—H21A	109.5
C14—C13—H13B	109.6	C18—C21—H21B	109.5
H13A—C13—H13B	108.1	H21A—C21—H21B	109.5
C13—C14—Cl2	112.5 (3)	C18—C21—H21C	109.5
C13—C14—H14A	109.1	H21A—C21—H21C	109.5
Cl2—C14—H14A	109.1	H21B—C21—H21C	109.5
C13—C14—H14B	109.1	C24—O5—C25	115.8 (2)
Cl2—C14—H14B	109.1	O5—C25—C26	105.7 (2)
H14A—C14—H14B	107.8	O5—C25—H25A	110.6
C16—C15—C24	120.9 (3)	C26—C25—H25A	110.6
C16—C15—S2	115.7 (2)	O5—C25—H25B	110.6
C24—C15—S2	123.2 (2)	C26—C25—H25B	110.6
C17—C16—C15	122.1 (3)	H25A—C25—H25B	108.7
C17—C16—H16	118.9	O6—C26—C25	107.5 (3)
C15—C16—H16	118.9	O6—C26—H26A	110.2
C16—C17—C22	117.1 (3)	C25—C26—H26A	110.2
C16—C17—C18	122.5 (3)	O6—C26—H26B	110.2
C22—C17—C18	120.4 (3)	C25—C26—H26B	110.2
C23—C22—C17	121.1 (3)	H26A—C26—H26B	108.5
C23—C22—H22	119.5	C26—O6—C27	110.1 (3)
C17—C22—H22	119.5	O6—C27—C28	109.5 (6)
C22—C23—C24	121.7 (3)	O6—C27—H27A	109.8
C22—C23—S1	117.0 (2)	C28—C27—H27A	109.8
C24—C23—S1	121.2 (2)	O6—C27—H27B	109.8
O5—C24—C23	120.1 (2)	C28—C27—H27B	109.8
O5—C24—C15	122.9 (2)	H27A—C27—H27B	108.2
C23—C24—C15	116.9 (2)	C27—C28—Cl1	114.0 (5)
C8—O7—C11	119.0 (2)	C27—C28—H28A	108.7
C12—O8—C13	112.3 (3)	Cl1—C28—H28A	108.7
O2—S1—O1	118.02 (14)	C27—C28—H28B	108.7
O2—S1—C23	108.83 (14)	Cl1—C28—H28B	108.7
O1—S1—C23	106.96 (13)	H28A—C28—H28B	107.6
O2—S1—C7^i^	106.76 (13)	C28'—C27'—H27C	109.2
O1—S1—C7^i^	107.68 (13)	C28'—C27'—H27D	109.2
C23—S1—C7^i^	108.26 (13)	H27C—C27'—H27D	107.9
O3—S2—O4	117.92 (14)	C27'—C28'—Cl1'	124 (3)
O3—S2—C9	108.63 (13)	C27'—C28'—H28C	106.2
O4—S2—C9	107.32 (13)	Cl1'—C28'—H28C	106.2
O3—S2—C15	106.68 (13)	C27'—C28'—H28D	106.2
O4—S2—C15	108.27 (13)	Cl1'—C28'—H28D	106.2
C9—S2—C15	107.62 (13)	H28C—C28'—H28D	106.4
			
C10—C5—C6—C7	0.0 (4)	C14—C13—O8—C12	−166.6 (4)
C4—C5—C6—C7	−179.6 (3)	C22—C23—S1—O2	−120.7 (2)
C5—C6—C7—C8	0.7 (4)	C24—C23—S1—O2	54.8 (3)
C5—C6—C7—S1^i^	175.6 (2)	C22—C23—S1—O1	7.8 (3)
C6—C7—C8—O7	−173.6 (2)	C24—C23—S1—O1	−176.6 (2)
S1^i^—C7—C8—O7	11.9 (4)	C22—C23—S1—C7^i^	123.6 (2)
C6—C7—C8—C9	−0.8 (4)	C24—C23—S1—C7^i^	−60.9 (3)
S1^i^—C7—C8—C9	−175.2 (2)	C8—C9—S2—O3	−51.8 (3)
O7—C8—C9—C10	173.2 (2)	C10—C9—S2—O3	123.9 (2)
C7—C8—C9—C10	0.2 (4)	C8—C9—S2—O4	179.7 (2)
O7—C8—C9—S2	−11.4 (4)	C10—C9—S2—O4	−4.6 (3)
C7—C8—C9—S2	175.6 (2)	C8—C9—S2—C15	63.4 (3)
C6—C5—C10—C9	−0.6 (4)	C10—C9—S2—C15	−120.9 (2)
C4—C5—C10—C9	179.0 (3)	C16—C15—S2—O3	−19.6 (3)
C8—C9—C10—C5	0.5 (4)	C24—C15—S2—O3	164.5 (2)
S2—C9—C10—C5	−175.2 (2)	C16—C15—S2—O4	108.3 (2)
O7—C11—C12—O8	−164.0 (3)	C24—C15—S2—O4	−67.7 (3)
O8—C13—C14—Cl2	−67.9 (4)	C16—C15—S2—C9	−136.0 (2)
C24—C15—C16—C17	−0.3 (4)	C24—C15—S2—C9	48.1 (3)
S2—C15—C16—C17	−176.3 (2)	C10—C5—C4—C1	6.4 (5)
C15—C16—C17—C22	−2.5 (4)	C6—C5—C4—C1	−174.1 (4)
C15—C16—C17—C18	176.6 (3)	C10—C5—C4—C3	−116.7 (4)
C16—C17—C22—C23	2.3 (4)	C6—C5—C4—C3	62.9 (4)
C18—C17—C22—C23	−176.9 (3)	C10—C5—C4—C2	126.6 (3)
C17—C22—C23—C24	0.7 (5)	C6—C5—C4—C2	−53.8 (4)
C17—C22—C23—S1	176.2 (2)	C16—C17—C18—C20	125.9 (4)
C22—C23—C24—O5	−179.1 (3)	C22—C17—C18—C20	−54.9 (4)
S1—C23—C24—O5	5.6 (4)	C16—C17—C18—C21	−110.2 (4)
C22—C23—C24—C15	−3.5 (4)	C22—C17—C18—C21	68.9 (4)
S1—C23—C24—C15	−178.8 (2)	C16—C17—C18—C19	6.0 (4)
C16—C15—C24—O5	178.8 (2)	C22—C17—C18—C19	−174.9 (3)
S2—C15—C24—O5	−5.5 (4)	C23—C24—O5—C25	−98.1 (3)
C16—C15—C24—C23	3.3 (4)	C15—C24—O5—C25	86.5 (3)
S2—C15—C24—C23	179.0 (2)	C24—O5—C25—C26	158.7 (3)
C9—C8—O7—C11	99.0 (3)	O5—C25—C26—O6	169.4 (3)
C7—C8—O7—C11	−88.4 (3)	C25—C26—O6—C27	170.1 (4)
C12—C11—O7—C8	−160.8 (3)	C26—O6—C27—C28	179.1 (5)
C11—C12—O8—C13	−174.6 (3)	O6—C27—C28—Cl1	−65.1 (9)

Symmetry codes: (i) −*x*+1, *y*, −*z*+3/2.

### Hydrogen-bond geometry (Å, °)

**Table:**

*D*—H···*A*	*D*—H	H···*A*	*D*···*A*	*D*—H···*A*
C11—H11A···O1^i^	0.99	2.48	3.232 (4)	133
C11—H11B···O3	0.99	2.51	3.103 (4)	118
C20—H20B···O1^ii^	0.98	2.57	3.377 (5)	139
C21—H21C···O8^iii^	0.98	2.60	3.462 (6)	146
C25—H25A···O2	0.99	2.58	3.099 (4)	113
C25—H25B···O4	0.99	2.45	3.217 (4)	134

Symmetry codes: (i) −*x*+1, *y*, −*z*+3/2; (ii) −*x*+1, −*y*+1, −*z*+1; (iii) *x*, −*y*+1, *z*−1/2.
